# Ipsilateral reexpansion pulmonary edema after drainage of a spontaneous pneumothorax: a case report

**DOI:** 10.1186/1752-1947-1-107

**Published:** 2007-09-29

**Authors:** Anna Conen, Ladina Joos, Roland Bingisser

**Affiliations:** 1 Division of Infectious Diseases and Hospital Epidemiology, University Hospital Basel, Switzerland; 2 Division of Pulmonary Medicine, University Hospital Basel, Switzerland; 3 Department of Emergency Medicine, University Hospital Basel, Switzerland

## Abstract

We report a case of ipsilateral reexpansion pulmonary edema occurring after the insertion of a chest tube in a patient with spontaneous pneumothorax. The patient received supplemental oxygen via a non-rebreather face mask to compensate for hypoxemia. 24 hours after the acute event, the patient recovered completely without residual hypoxemia. Reexpansion pulmonary edema after the insertion of a thoracic drainage for pneumothorax or pleural effusion is a rare complication with a high mortality rate up to 20%. It should be considered in case of hypoxemia following the insertion of a chest tube. The exact pathophysiology leading to this complication is not known. Risk factors for reexpansion pulmonary edema should be evaluated and considered prior to the insertion of chest tubes. Treatment is supportive.

## Introduction

In 1958, reexpansion pulmonary edema (REPE) was first described by Carlson [1]. Since this first description several case reports and case series have been published. REPE is a rare complication occurring after the insertion of a chest tube for pneumothorax or pleural effusion. The incidence ranges between 1–14%, as described in 2 retrospective analyses from 1991 [2] and 1996 [3]. REPE can occur on the ipsi- or contralateral side, can be bilateral and can even be asymptomatic [3-7].

The exact pathophysiology for this complication is unknown. Mechanical distress on the alveoli or oxygen radicals might be a contributing factor [8,9]. Oxygen radicals are produced during the hypoxemia in the collapsed lung. Moreover, the activity of different cytokines such as interleukin 8 and monocyte chemoattractant protein 1 (MCP-1) [10], or the activity of xanthine oxidase [11] have been implicated in the pathogenesis of REPE. In the literature, several risk factors have been associated with REPE: younger age (<40 years), longer duration of lung collapse (>4 days), large pneumothorax (>30% of a single lung) and timing of lung reexpansion [2,8,12]. In a case series in 1988, a mortality rate as high as 20% has been described [8], which was also cited by Sherman in 2003 [13].

We here present a case of ipsilateral reexpansion pulmonary edema occurring after the insertion of a chest tube in a patient with spontaneous pneumothorax. The case is illustrated with unique radiological figures as well as a figure showing the course of the oxygen saturation in correlation to the oxygen administered over the initial 12 hours.

## Case presentation

**Figure 1:**
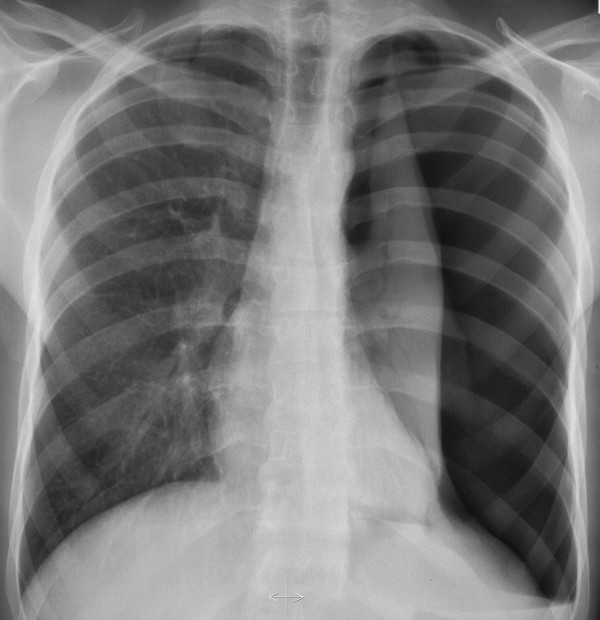
Chest radiography with left lung collapsed and slight mediastinal shift.

**Figure 2:**
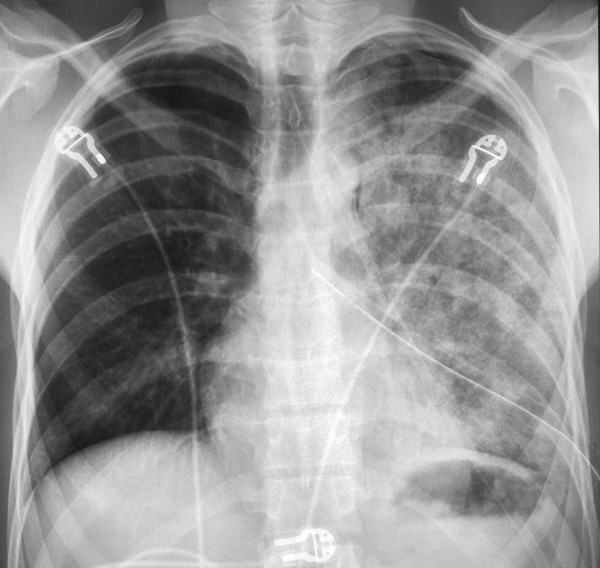
Chest radiography with left-sided pulmonary edema.

A 25 year old male patient presented to our emergency department 24 hours after the onset of a sudden left-sided inspiratory chest pain without any further associated symptoms. His medical history was unremarkable except for a smoking history of nine pack years. The patient was in good general health with normal vital signs, including respiratory rate (16/min) and oxygen saturation (98% when breathing ambient air). Physical examination was normal except for diminished breath sounds on the left side, as were laboratory values. Chest X-ray confirmed the clinical suspicion of a left sided spontaneous pneumothorax with slight mediastinal shift as a possible sign of a beginning tension pneumothorax (figure 1). A chest tube was inserted in local anesthesia in the left midaxillary line without complication. A negative pressure of 20 cm H2O was applied. Ninety minutes after the insertion of the chest tube with drainage and relief of symptoms, the patient complained of dyspnea and increasing pain in the left chest. Chest auscultation revealed left-sided inspiratory crackles. Oxygen saturation decreased to 85% despite the addition of oxygen (initially 2 l/min via nasal cannula). Later oxygen saturation stabilized at 90% with 12 l/min oxygen via a non-rebreather face mask. The arterial blood gas analysis under 12 l/min oxygen showed hypoxemia: pO2 7.9 kPA (>10.7 kPA), pCO2 5.5 kPA (5–5.5 kPA), pH 7.36 (7.38–7.42), bicarbonate 23 mmol/l (21–26 mmol/l) and oxygen saturation of 90%. A second chest X-ray demonstrated an expanded left lung, but also an ipsilateral pulmonary edema (figure 2).

**Figure 3:**
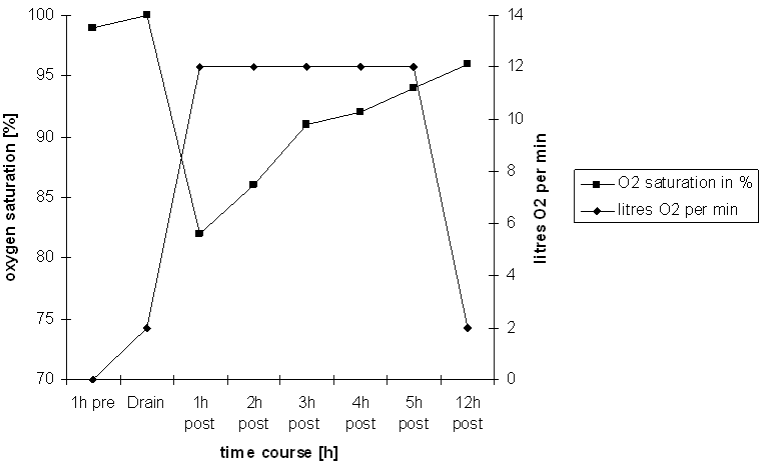
Surveillance of oxygen saturation in correlation to the oxygen administered.

**Figure 4:**
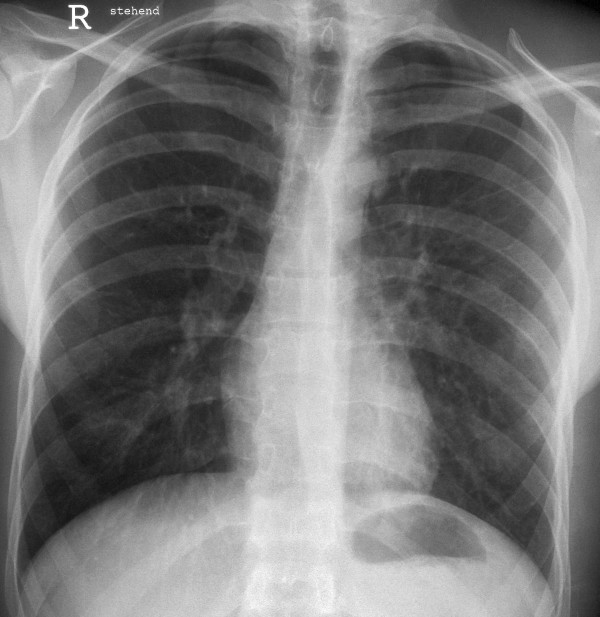
Chest radiography with reexpanded left lung and decreased pulmonary edema.

The patient stabilized under continuous oxygen (12 l/min via a non-rebreather face mask) with his oxygen saturation steadily increasing. No other treatment was necessary. The patient was transferred to the medical floor where the negative pressure on the thoracic drainage was continued. The patient's further hourly clinical observation was unremarkable over the course of 12 hours (figure 3). Finally, the patient was in a good condition without requiring any additional oxygen. The chest drainage was removed when no more air leakage was detected on day 2 of hospitalization. A third chest X-ray 12 hours after the removal of the chest drainage showed a completely reexpanded left lung and a decreased pulmonary edema (figure 4).

The patient was discharged without any further complications. The clinical follow-up for 3 months was unremarkable. During follow-up no CT scan of the chest was performed, because the conventional chest X-ray was completely normal and also to prevent the young patient from additional radiation, even though the requirement of only low radiation doses for the quantification of pulmonary emphysema has been shown [14]. Therefore, in case of any subtle abnormality on the conventional chest X-rays, CT scans are warranted in order to search for bullous lung diseases.

## Discussion

We report a case of a REPE after the insertion of a chest tube for a spontaneous pneumothorax. Our patient suffered from this rare complication but showed a benign clinical course. He was successfully treated with the sole administration of oxygen via a non-rebreather face mask. His oxygen saturation improved steadily and normalized after 12 hours. Usually REPE is self limited and can even be asymptomatic [3]. However, a mortality rate as high as 20% has been described [8,13]. This rare complication and the treatment of REPE should be known by clinicians and particularly considered when a patient's condition declines after initial amelioration [7]. Treatment is supportive, mainly consisting of the administration of supplemental oxygen and morphine if needed [3]. Occasionally, non-invasive continuous positive pressure ventilation or even mechanical ventilation are required to provide adequate oxygenation [6,15]. In severe cases – i.e. failure of mechanical ventilation – differential lung ventilation might be a treatment alternative [16].

Some risk factors – as described in the literature [2,8,12] – were present in our patient: younger age, large pneumothorax and rapid reexpansion by the administration of negative pressure. The presence of these risk factors does not predict an adverse outcome with certainty but should guide the clinician to a more cautious procedure in order to prevent REPE. In patients with these risk factors, the administration of initial negative pressure should be avoided [17]. In our patient, the relevance of these risk factors was possibly underestimated or overlooked.

Summarizing, REPE is a rare but severe complication after the insertion of a chest tube for spontaneous pneumothorax and can be lethal. Slow reexpansion of the pneumothorax and minimal or no negative pressure should be applied if risk factors are present in order to avoid this life-threatening complication.

## Conclusion

- Reexpansion pulmonary edema is a rare complication with high mortality rate occurring after thoracic drainages for pneumothorax or pleural effusions.

- Clinicians should be familiar with this complication and the associated risk factors.

- Treatment is symptomatic.

## Abbreviation list

REPE – Reexpansion pulmonary edema

## Competing interests

The author(s) declare that they have no competing interests.

## Authors' contributions

AC summarized the case and designed and drafted the manuscript. LJ participated in the design and coordination of the manuscript. RB initialized the case report and helped to draft the manuscript. All authors read and approved the final manuscript.
